# Age-Related Difference in the Relationship Between Age Stereotypes and Health Behavior in Midlife Koreans

**DOI:** 10.1093/geroni/igaa057.1448

**Published:** 2020-12-16

**Authors:** Hyun-E Yeom, Eunyoung Park, Misook Jung

**Affiliations:** 1 Chungnam National University, Daejeon, Taejon-jikhalsi, Republic of Korea; 2 Chungnam National University, Daejeon, Republic of Korea

## Abstract

Aging stereotypes about health may affect the interpretation of health-related changes in midlife. The purposes of this study were to examine the influence of age-related stereotypic beliefs on health behavior linked to health status and stress and to investigate whether the relationship differs by age. This is a cross-sectional study using a convenience sample of 446 Korean middle-aged adults (mean age=50.7). Data were analyzed using the PROCESS macro approach of SPSS. Age-related stereotypic beliefs affected health behavior through the evaluation of health status (Index=-.006, 95% CI [-.002, -.001]) and related stress (Index=-.002, 95% CI [-.004, -.001]). The relationship between age-related stereotypic beliefs and health status significantly differed by age (β= -.02, p=.004), indicating that individuals with stronger age-related stereotypic beliefs tended to report poorer health status as they aged. With regard to the age difference, the influence of age-related stereotypic beliefs affecting health behavior through health status and related stress became relatively stronger when midlife individuals became older. The findings of this study highlight the importance of carefully assessing age-related beliefs based on consideration of age-related differences in middle-aged individuals. Developing psychoeducational strategies to modify the negative and erroneous aging-related beliefs and to improve health behavior is warranted.

